# Management of a Complex Dentoalveolar Traumatic Injury with Multiple Avulsions

**DOI:** 10.1155/2021/2373785

**Published:** 2021-06-28

**Authors:** Anita Thakur, Seema Thakur

**Affiliations:** Department of Pediatric and Preventive Dentistry, H.P Government Dental College and Hospital Shimla, Himachal Pradesh, India

## Abstract

**Background:**

Dentoalveolar trauma is a major cause of tooth loss in children. Avulsion, luxation, crown, and root fracture are the injuries to primary and permanent dentition. The incidence of trauma for maxillary anterior teeth ranges for 4%-91%. Many case reports have been published regarding the treatment of trauma to anterior teeth; however, case reports comprising multiple avulsions including canines and premolars are rare in literature.

**Method:**

After mouth rinsing was done with 2% betadine solution, the luxated teeth numbers 31 and 42 were repositioned into the tooth socket and were secured with the composite resin-wire splint. Tooth number 32 was extracted because it was disarticulated from the socket, and the socket was disrupted because of the alveolar fracture. The maxillary avulsed teeth could not be reimplanted because of the alveolar socket damage which was due to the alveolar bone fracture.

**Results:**

The patient was reevaluated for the removable prosthesis in recall visits; the patient was well adapted to the appliance with no complaints regarding mastication and speech. The patient was advised to report periodically for further adjustments in the prosthesis and for radiographic evaluation.

**Conclusions:**

This case report includes proper history taking, diagnosis, and treatment of a complex dentoalveolar trauma along with short-term prosthetic rehabilitation for improvement of aesthetics, phonetics, and mastication of growing child.

## 1. Background

Dentoalveolar trauma is one of the main causes of tooth loss in pediatric population. The trauma can lead to tooth loss via variety of ways like untreated avulsion, root resorption, or extraction due to acute injury [1]. These injuries of the teeth and supporting structures to the children can pose a major challenge to the practitioner because of the dynamic state of occlusal development [2–4]. Avulsion, luxation, crown, and root fracture are the injuries to primary and permanent dentition. Injury to deciduous teeth may damage the underlying permanent tooth; however, if trauma occurs to the permanent teeth, it may compromise their long-term prognosis [3]. The most common cause of dental injury was fall, followed by sports [5]. The incidence of trauma for maxillary anterior teeth and premolars is reported in between 4%-91% and 0.6%, respectively. The incidence reported for luxation injury is 26%, for alveolar fracture 5.5%, for soft tissue injury 47%-58%, and 4%-22% for avulsion in the literature [6–9]. This great variation in reporting rates could be attributed to many factors including type of study, trauma classification, study size and population, geographical area, and different cultural behaviours [10]. Many case reports have been published describing the treatment of avulsed permanent incisors but the treatment following multiple avulsions including canines and premolars has been rarely found in the literature. This case report includes history taking, diagnostic considerations, and immediate treatment plan followed by transitional prosthetic rehabilitation for young child.

## 2. Case Report

An 11-year-old male child was referred to the Department of Pediatric and Preventive Dentistry, Himachal Pradesh Govt Dental College and Hospital (HPGDC) Shimla, India, following severe dentoalveolar trauma along with soft tissue injuries. The parents reported that when the child was returning from the school, he got hit by a motorcycle and fell on the ground. At the time of the injury, the child spat out many permanent and deciduous teeth of both the arches. The patient had two episodes of vomiting and history of oral bleeding at the time of the traumatic injury. There was no history of loss of consciousness, seizures, and bleeding from ear and nose. The mother collected the knockedout teeth in an empty box, and the child was taken to the nearby regional hospital. The soft tissue wounds were debrided, and lacerated wounds were sutured by the physician attending the child, and the patient was referred to Indira Gandhi Medical College and Hospital (IGMC), Shimla. The patient was attended in the emergency department of the medical hospital and further referred to HPGDC for additional care because of the severity of dentoalveolar trauma.

**Figure 1:**
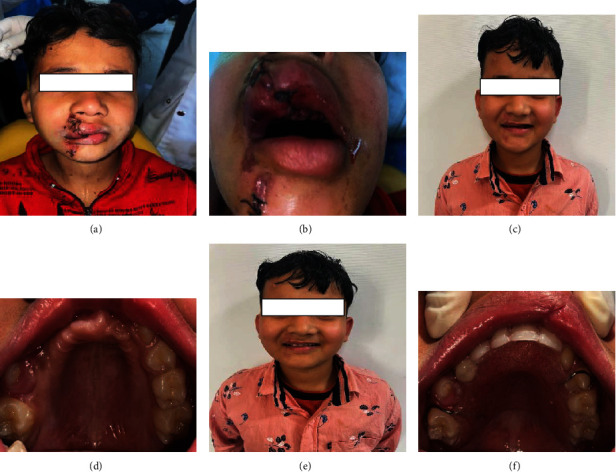
(a–f) Preoperative and postoperative (installation of removable prosthesis in the maxillary arch) photographs of the pat.

The patient arrived in the Department of Pediatric and Preventive Dentistry 24 hr after the trauma. On extraoral examination, the face was apparently symmetrical with diffuse swellings present with respect to the upper lip and chin regions. Sutures were present on the right side of upper lip extending beyond the vermilion border and chin region. Mandibular movements were restricted, and tenderness on palpation was present with respect to chin region and bilateral lower molar region (Figure 1(a)).

**Figure 2:**
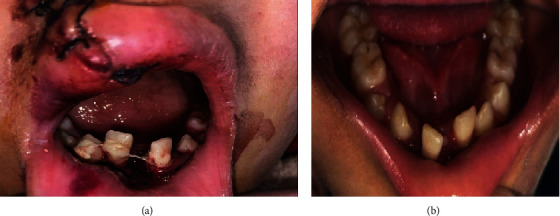
(a) Composite wire splint and (b) removal of splint after 4 weeks.

**Figure 3:**
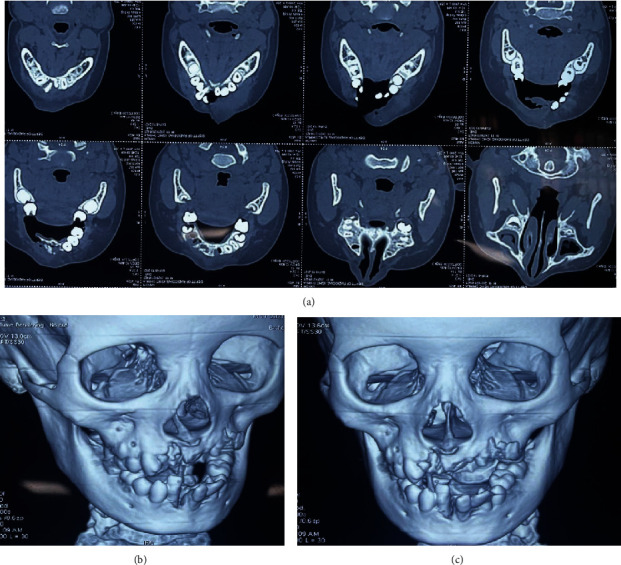
(a–c) Computed tomography (CT) scan showing multiple avulsions along with alveolar bone fracture.

**Figure 4:**
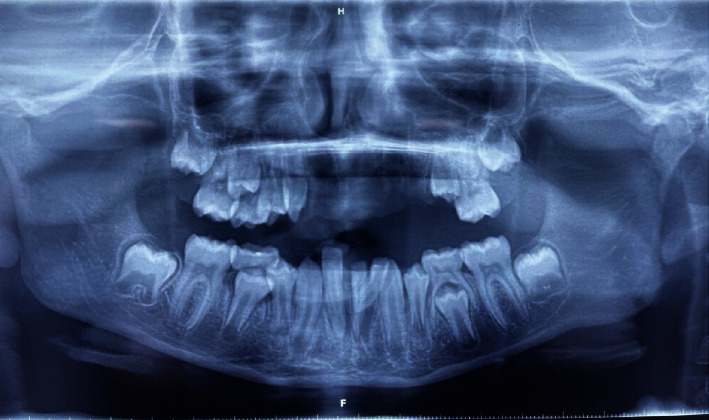
Radiograph showing multiple avulsions of the teeth.

Intraoral examination revealed deranged occlusion with inadequate mouth opening, and sutures were present on the right side of the upper and lower lips. Multiple bruises and lacerations were also visible. There was an avulsion of teeth numbers 11, 12, 21, 22, 23, 24, 65, and 41. Tooth number 32 was disarticulated from the tooth socket along with the fracture of labial and lingual cortical plates. Teeth numbers 31 and 42 were luxated from the tooth socket (Figures 2(b) and 2(c)). Extensive intraoral damage to the soft tissue was noted in the maxillary area with multiple abrasions and lacerations. Sutures were present in the maxillary alveolar ridge area which was placed after compressing and approximating the tooth socket area in the regional hospital (Figure 1(b)). The dentoalveolar fracture with multiple avulsions was clearly evident from the computed tomography (CT) scan (64 Slice MDCT scanner Light Speed VCT-XTE GE Healthcare, Grandview Blvd. Waukesha, WI, U.S.A) of the patient which was performed in the IGMC (Figures 3(a)–3(c) and 4).

An informed verbal and written consent was taken from the patient and parents, respectively. The patient was taken to the minor operating theatre for the treatment under local anaesthesia. The mouth rinsing was done with 2% betadine solution (Cipladine, Cipla Ltd. Mumbai, India) before the procedure. The parents were informed about the risks, benefits, and poor prognosis of the proposed treatment. The luxated teeth numbers 31 and 42 were repositioned into the tooth socket and were secured with the composite resin-wire splint [1] (Figure 2(a)). Tooth number 32 was extracted because it was disarticulated from the socket, and the socket was disrupted because of the alveolar fracture (Figures 3(a)–3(c)). The maxillary avulsed teeth could not be reimplanted because of the alveolar socket damage which was due to the alveolar bone fracture (Figures 3 and 4).

Oral hygiene instructions were given, and the patient was discharged to the care of his parents. The parents were asked to keep the teeth as clean as possible, and a chlorohexidine mouthwash was given for rinsing. Amoxicillin with clavulanic acid (375 mg t.i.d.) and acetaminophen was prescribed as discharge medicine. Patient was advised to take soft diet for 10-14 days.

After 10 days, satisfactory postoperative healing was there and the sutures were removed. The patient was followed for 2, 4, and 6 weeks. The healed maxillary and mandibular arches can be appreciated in Figures 1(d) and 2(b). After 4 weeks, the splint was removed as per the Guidelines of International Association of Dental Traumatology (Revised in June 2020) [11] and American Associations of Endodontists [12]. Maxillary and mandibular arch alginate (Plastalgin, Septodont Healthcare India Pvt. Ltd. Raigad, Maharashtra, India) impressions were taken after 6 weeks of soft tissue healing to fabricate an acrylic prosthesis (Figures 1(c)–1(f)). Teeth numbers 31 and 42 were asymptomatic clinically as well as radiographically with intact lamina dura. The patient was reevaluated for the removable prosthesis in recall visits, and the patient was well adapted to the appliance with no complaints regarding mastication and speech. The patient was advised to report periodically for further adjustments in the prosthesis in case of permanent tooth eruption and for radiographic evaluation.

## 3. Discussion`

Traumatic dental injury is not a result of a disease but can be a consequence of many factors that will get accumulated through entire life if not treated properly [13]. Emotional preparation of the child is required prior to the dental treatment. Trauma can increase patient's apprehension towards the treatment. However, extensive trauma to dentoavleolar part requires immediate intervention and treatment for the stability of child's physical and emotional integrity [14]. It also prepares the child and the family for an expensive and extensive restorative future [2]. This case report involved extensive lesions of extraoral soft tissue over lip and chin area, teeth, and alveolar bone which are rarely seen in dental practice as most of the dental avulsions affect only a single tooth [14–16]. The most common teeth involved in the dental trauma were maxillary incisors as compared to mandibular incisors because any blow to the mandibular teeth was dissipated due to nonrigid connection of the mandible to the base of cranium [13].

According to the studies in the literature, boys had suffered more traumatic injuries than girls which could be attributed to the behavioural factors as boys tend to be more energetic and inclined towards outdoor activities and contact sports [5]. Severe damage was occurred to the left half of the maxilla following loss of multiple permanent teeth and fracture of the alveolar bone. The permanent maxillary anterior teeth and premolars could not be reimplanted because of the damage to the alveolar bone [2]. The deciduous tooth was also not reimplanted because of its proximity to permanent tooth germ, and it can cause significant alteration to the succedaneous tooth [17]. It may result in worst consequences like dilacerations, enamel hypoplasia, imapctions, or deviation from the normal path of eruption. Some studies have also reported radicular cyst associated with the replanted primary tooth [18]. Also, it should be noted that the dry storage time beyond 60 minutes had a strong relationship with replacement resorption [19]. Poor prognosis of the treatment done can develop because of periodontal ligament death, pulp necrosis which leads to ankylosis, internal resorption, replacement resorption, and eventually the loss of tooth [19]. The main reason for loss of replanted teeth is resorption. The risk of early resorption is increased in teeth which are having additional damage or contamination or which are kept in dry extraoral conditions for more than 15 min. The teeth, whose periodontal ligaments have dried, destroyed, and removed from the root surface, result in replacement resorption and get fused with the alveolar bone [20].

After healing of the alveolar tissue, maxillary prosthesis was given for restoration of aesthetics and phonetics of the young child [2, 14] (Figures 1(d)–1(f)). The mandibular teeth were splinted with wire composite splint [1–3, 21]. This nonrigid splint was removed after 4 weeks [19, 21] as “splinting for 4 weeks was shown to influence the healing pattern” [19]. The dental trauma healing mainly depends upon firstly on repositioning and splinting and secondly, the infection prevention. Antibiotic prophylaxis was mainly used for controlling soft tissue infections; however, its role to influence the wound healing has not been found in traumatic dental injuries. In clinical studies, application of splints in some cases can add extra damage to the periodontal and pulpal tissue [22].

There are many consequences after early loss of permanent teeth, and the indication of a prosthetic rehabilitation with miniscrew-assisted provisional restorations in aesthetic zone may be an alternative for an effective treatment. This technique can also be used in growing patients as miniscrews have been demonstrated to have excellent mechanical properties and can support the patient until adult age, when conventional implants can be placed [23, 24]. Along with restoring aesthetics, it will also help in avoiding any alterations in phonetic and masticatory functions. However, it prepares the child psychologically to accept and collaborate with the proposed treatment in order to be socially reintegrated [14].

Within the limitation of the present case report, it was a matter of trying what was believed to be the best approach for this patient. However, reviewing the literature, it is apparent that the approach to deal with these aspects of trauma needs constant update, which is based on our clinical decisions.

## 4. Summary

To summarize, knowledge and ability of the professional are put to test while handling a pediatric traumatic emergency. Acute dental treatment is an important aspect following dental traumatic injuries. Awareness regarding tooth storage media and preventive education programs should be instituted at the level of parents (including pregnant mothers), school teachers, and common people so that they can contribute to better prognosis of traumatized tooth. Another important aspect is to improve knowledge among general dentists regarding aetiology, diagnosis, treatment, and complications of trauma.
